# Influence of electrodeposition parameters on the fabrication of Ni–Co/SiC + TiN composite films through pulse current electrodeposition

**DOI:** 10.1038/s41598-024-64083-8

**Published:** 2024-06-07

**Authors:** Tianqi Cui, Mengyu Cao, Huaxing Li, Yu Zhang, Kedi Jiang

**Affiliations:** College of Mechanical Science and Engineering, Northeast Petroleum University, Daqing, 163318 China

**Keywords:** Ni–Co/SiC + TiN, Composite film, Pulse current electrodeposition, Pulse frequency, Duty cycle, Materials science, Nanoscience and technology

## Abstract

In this investigation, pulsed current electro-deposition (PCE) was used to prefabricate Ni–Co/SiC + TiN composite coatings (NCSTCCs) on mild steel surfaces. The research focused on the influence of two electrodeposition parameters, pulse frequency (PF) and duty cycle (DC), on NCSTCF features including microscopic surface morphology, crystal orientation, grain size, microhardness, SiC and TiN nanoparticles (NPs), deposition quantity, and corrosion resistance properties. The results indicated that NCSTCCs produced under a 10% DC showed minimal SiC and TiN contents with a percent volume of just 5.6 v/v% and 5.4 v/v% respectively under the fixed condition of 60 Hz PF. However, the three-dimensional surface diagram indicated that the Ni–Co/SiC + TiN composite film deposited at 50% DC and 10 Hz PF displayed the highest SiC and TiN contents (11.6 v/v% and 11.7 v/v%) among all the films. Furthermore, NCSTCCs deposited under 50% DC and 10 Hz PF had peak microhardness at 667.4 kg/mm^2^, while the composite film achieved a microhardness of 514.1 kg/mm^2^ when prepared using 10% DC and 60 Hz PF. Moreover, when the DC and PF were at 50% and 10 Hz respectively, the Ni–Co/SiC + TiN composite film presented the maximum charge transfer resistance (4915.7–4927.2 Ω·cm^2^), indicating an excellent corrosion resistance.

## Introduction

It is widely recognized that ceramic nanoparticles (CNPs) have garnered significant attention from scientists globally due to their exceptional mechanical and chemical properties^1–3^. Generally, CNPs with high wear resistance, high hardness, and excellent corrosion resistance are deposited into metal substrates as reinforcing phase particles, such as TiO_2_, SiC, TiN, and WC to improve the hardness, oxidation resistance, wear, and corrosion performance of metal matrix composites^4–6^. The unique property of Ni-based SiC composite coatings is their ability to improve corrosion resistance and tribological abilities on the surface of metal pure Ni substrates. A few studies have found that replacing pure nickel substrate with Ni–Co alloy can improve the performance of Ni-based SiC composite coatings^7–9^. Currently, numerous researchers have examined corrosive performance for NCSTCCs, whereas the anticorrosion properties of these films have received less attention^10–13^.

To date, single nanoparticle composite coatings have been studied by many researchers. Zhang et al*.* prepared Ni–SiC nanocomposites on the surface of Q235 steel and studied the mechanical and electrochemical properties of the composite under different magnetic field strengths^5^. Liu et al*.* used ultrasonic electrodeposition to prepare Ni–TiN nanofilm coatings and investigated the microstructure and mechanical properties of the prepared film samples^14^. However, as the performance of single nanoparticle composite coatings increasingly fails to meet the demand for various high-performance requirements, combining two types of nanoparticles for their synergistic effects is a feasible approach. The combination of SiC and TiN nanoparticles is particularly advantageous because SiC improves wear resistance and oxidation resistance due to its high hardness, while TiN contributes to excellent corrosion resistance and enhanced hardness. Together, these nanoparticles can synergistically reinforce the mechanical and electrochemical properties of the composite, resulting in superior tribological and anti-corrosion performance compared to single-nanoparticle composites. Furthermore, adding two types of nanoparticles allows for better customization of the composite film’s properties to meet specific application needs. This study introduces a novel approach by utilizing a dual nanoparticle reinforcement strategy to enhance both mechanical and electrochemical properties, markedly improving wear resistance, oxidation resistance, and corrosion protection.

Pulsed current electrodeposition (PCE) is well known as one of the processing techniques used for developing NCSTCCs, having advantageous ease, high efficiency, and low cost^5,14,15^. Several parameters, including current density, duty cycle (DC), and pulse frequency (PF), can have a considerable impact on the overall anti-corrosion potential of deposited Ni–Co/SiC + TiN composite coatings during PCE preparation. Xia et al*.* studied the influence of the law of current density parameters on the performance of Ni-based ceramic composite coatings in previous laboratory reports^16^. Our research not only further investigates these parameters but also introduces new insights into the effect of dual nanoparticle systems on the microstructure and mechanical integrity of the coatings. In the current study, NCSTCCs were successfully deposited on the mild steel (MS) surface using the PCE process, with the goal of enhancing MS corrosion resistance. Furthermore, the effect of the two electrodeposition parameters, DC as well as PF, on the grain orientation, surface morphology, crystal size, microhardness, number of embedded SiC and TiN NPs, and corrosion resistance composite coatings deposited by PCE was investigated. Scanning electron microscopy (SEM) together with X-ray diffraction (XRD) respectively examined surface morphology together with crystalline orientation, and energy-dispersive X-ray spectroscopy (EDXS) determined SiC and TiN NPs quantities deposited in NCSTCCs. Besides, the microhardness and anti-corrosive properties for NCSTCCs were also evaluated.

## Experimental section

An epoxy sealer was used to encapsulate the exterior surface of a mild steel specimen used as the electrode, leaving only a 20 mm × 20 mm working area for deposition. Prior to pulse current electrodeposition, the specimen's outer surface was polished separately with 200, 600, 800, and 1200 grit sandpapers, and then rinsed with 8 g/L Na_2_CO_3_ and 8 g/L NaOH mixture at 25 °C. The specimens were immersed for 15 s through 1:1 HCl mixture. The PCE plating mixture had the following ingredients in the following proportions: 75 g/L CoSO_4_·7H_2_O, 200 g/L NiSO_4_·7H_2_O, 45 g/L NiCl_2_·6H_2_O, 30 g/L sodium citrate, 10 g/L SiC NPs with a diameter of 40 nm, 10 g/L TiN TPs with a diameter of 40 nm, and 20 g/L boric acid.

SiC nanoparticles (NPs, purity: 99.98%) and TiN nanoparticles (NPs, purity: 99.98%) were suspended in a plating mixture prior to PCE, and the plating mixture was ultrasonically agitated with 250 W power for 16 min to prevent SiC and TiN NPs agglomerating. PCE tests included 10–70% DC variation intervals, together with 10–60 Hz PF variation intervals. The deposition pulse (ton) ranged from 1.67 to 50 ms, while the pause duration (toff) was 15–50 ms. Table 1 shows detailed process parameters used to produce NCSTCCs using the PCE method.

**Table 1:** Plating parameters for fabricating Ni–Co/SiC + TiN composite coatings.

Plating condition	Parameters
Current density (A/dm^2^)	4
Pulse frequency (Hz)	10, 30, 60
Duty cycle (%)	10, 30, 50, 70
pH	4.6
Bath temperature (°C)	50

Quanta FEG450 SEM was used to comprehensively examine the Ni–Co/SiC + TiN composite film’s surface morphology. Based on this, the total amount of SiC and TiN NPs presents in the films was determined using a combination of EDXS techniques. It was determined that the average thickness of the composite coating was ~ 70 μm. A Philips D5000 X-ray diffractometer was used to determine grain size and crystallographic orientation. For the measurement parameters, Cu Kα rays were selected, the scanning range 2θ value was varied from 10° to 80°, while scanning speed was placed at 0.03°/s. The average NiCo size (*D*) was estimated using the Debye–Scherrer formula.

Vickers DT-950 microhardness analyzer was used to determine the microhardness of composite coatings. A constant load of 100 g was applied for 15 s to obtain the readings. The microhardness of each film sample was determined by taking the average of five measurements. In a mixture of similar composition to seawater (NaCl 25 g/L, MgSO_4_ 3 g/L, MgC1_2_ 2 g/L, CaC1_2_ 1 g/L) while anti-corrosive function for prepared NCSTCCs were measured using electrochemical impedance spectroscopy (EIS). Through CS350 electrochemical platform having sample (MS coated NCSTCCs) working electrode, a platinum sheet (reverse electrode), together with standardized saturated-mercury electrode (reference electrode), EIS of the coatings was determined. Before the EIS test, the sample was immersed in the seawater-like mixture for over 30 min, long enough for the corrosion potential to stabilize. The interference level was 10 mV, while identifying frequency spanned 10 mHz to 10 kHz to determine the EIS.

## Results and discussion

### Surface morphology

**Figure 1:**
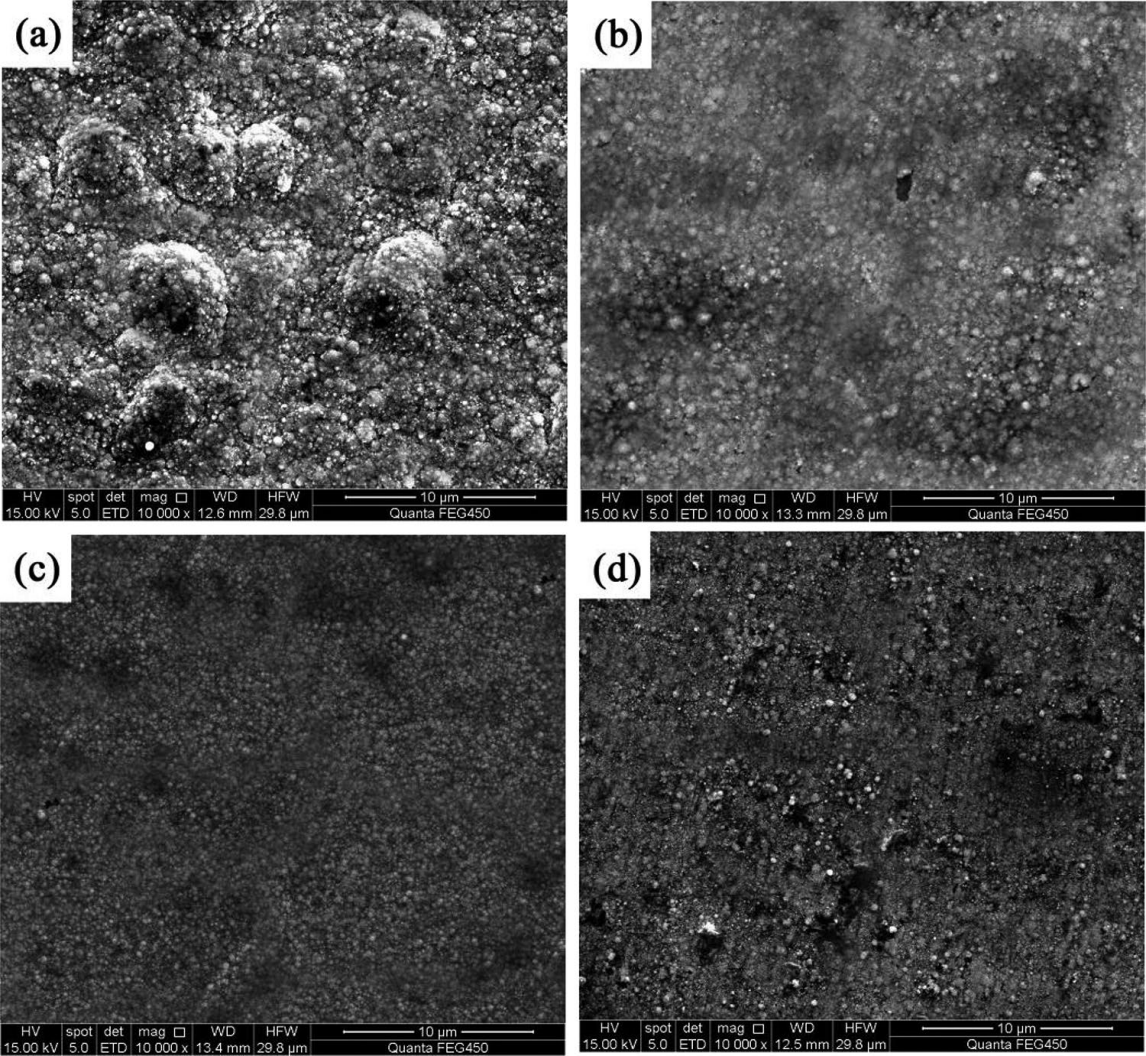
NCSTCCs surface morphologies obtained with various DCs at 10 Hz of fixed PF: (**a**) 10%, (**b**) 30% (**c**) 50%, and (**d**) 70%.

**Figure 2:**
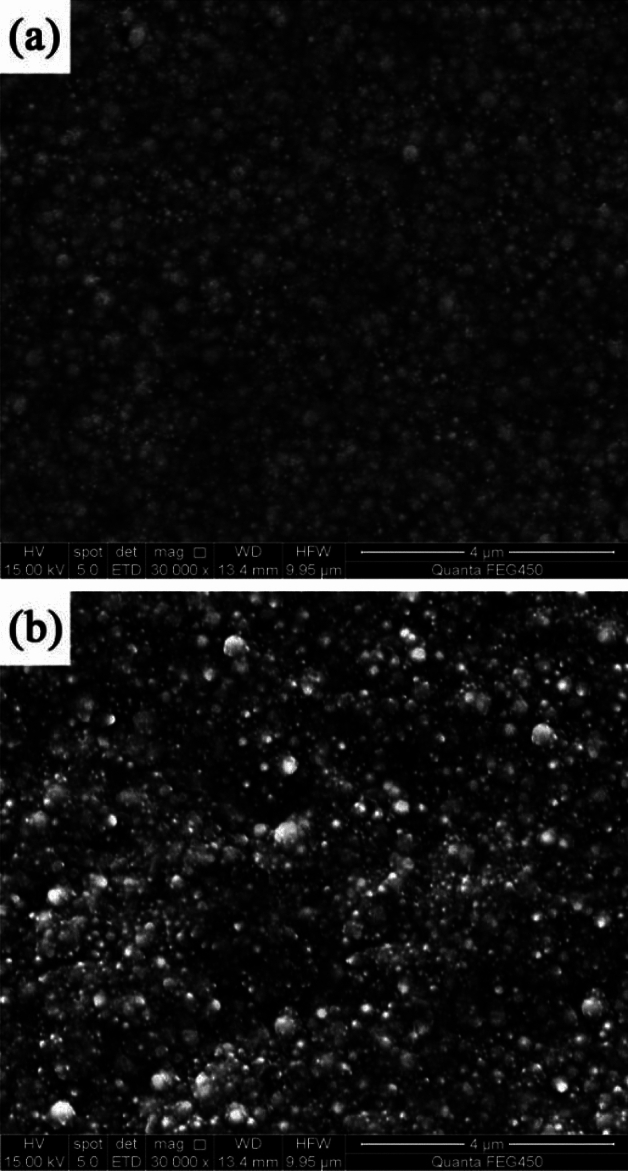
Amplified SEM pictures of NCSTCCs obtained at different duty cycles: (**a**) 50% and (**b**) 70%.

Figure 1 shows the surface morphologies of NCSTCCs developed at a fixed PF of 10 Hz with varying DCs. The films were found to have cauliflower-like globular morphological structures. When the DC was increased to 50%, the cauliflower-like structural morphology changed to nodular morphology, in which the cauliflower-like grains became small and fine. Furthermore, the surface morphological characteristics of the composite coatings derived at 50% and 70% DCs are exhibited in Fig. 2. The magnification of the surface morphology of those composite coatings shown in Fig. 2 was comparatively higher than that of Fig. 1. During the electrodeposition process, it was discovered that NCSTCCs obtained at 50% DC had a finer and harder surface morphology than those derived at 70%.

**Figure 3:**
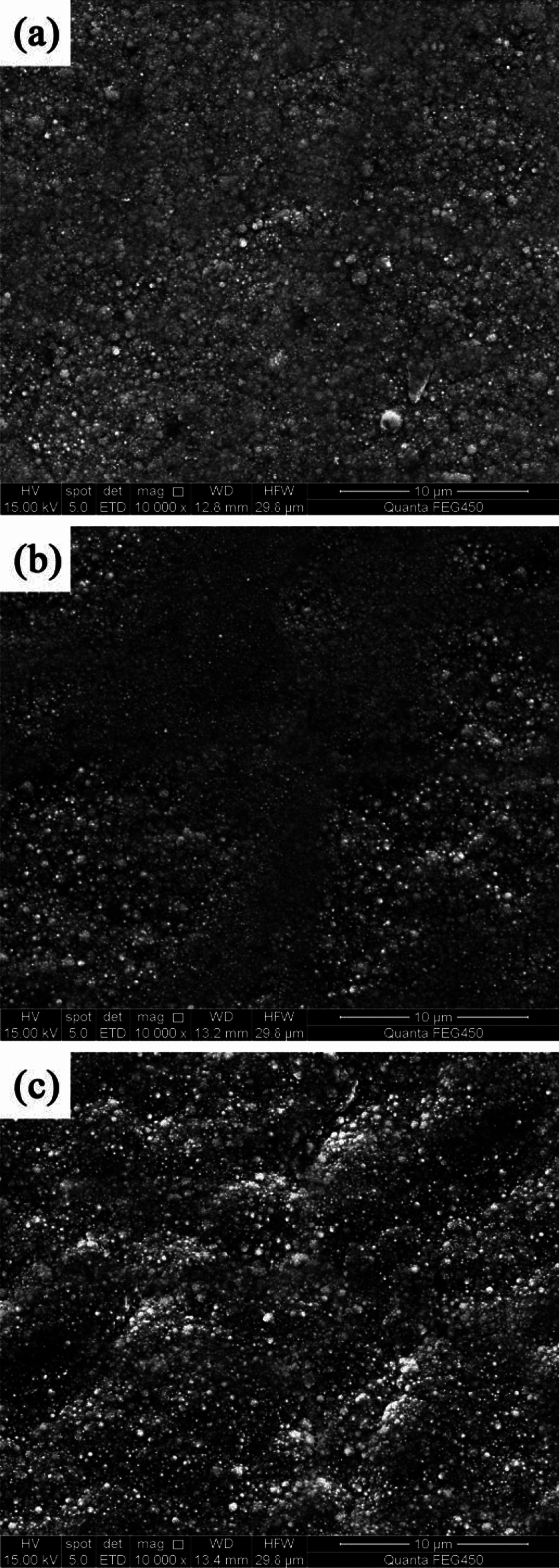
NCSTCCs surface morphologies obtained at 50% DC and different PFs: (**a**) 10 Hz, (**b**) 30 Hz, and (**c**) 60 Hz.

**Figure 4:**
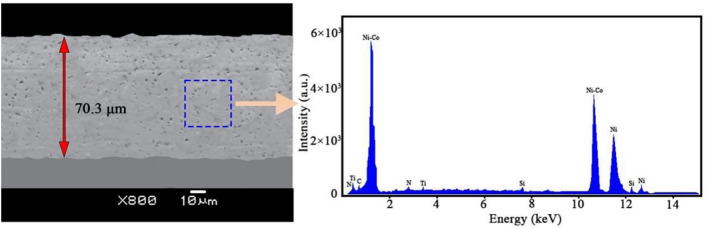
Superficial characteristics for SiC and TiN NPs embedded within NCSTCCs obtained at 50% DC and 10 Hz PF.

Different pulse frequencies with a constant DC of 50% resulted in distinct surface morphologies for NCSTCCs (Fig. 3). At 10 Hz PF, the structure property of the film was primarily nodular in shape, with essentially minimal presence of large grains. The structure of the film changed to cauliflower-like as the PF gradually increased. The amount and size of large grains in the developed film increased as the PF was increased towards 60 Hz. Figure 4 shows a cross-section of the composite film and shows the surface characteristics of the SiC and TiN NPs embedded in the ncstcf. It can be seen from the figure that SiC and TiN particles exist not only on the surface of the film, but also in the film, and the dispersion is high. The thickness of the film is 37.2 μm, in which the SiC and TiN nanoparticles are aggregated to form particle clouds and implanted into the Ni–Co grains as the second phase. This result is similar to that of Liu et al.^17^.

### SiC deposition content

**Figure 5:**
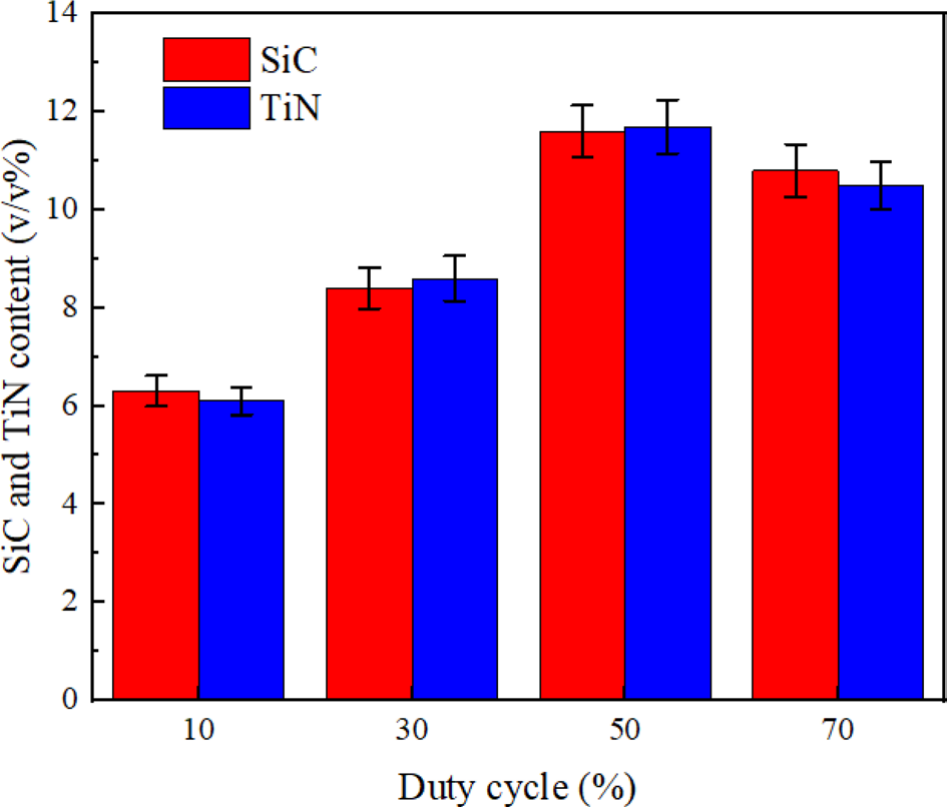
DC influence upon SiC and TiN NPs levels embedded in NCSTCCs obtained at 10 Hz PF.

**Figure 6:**
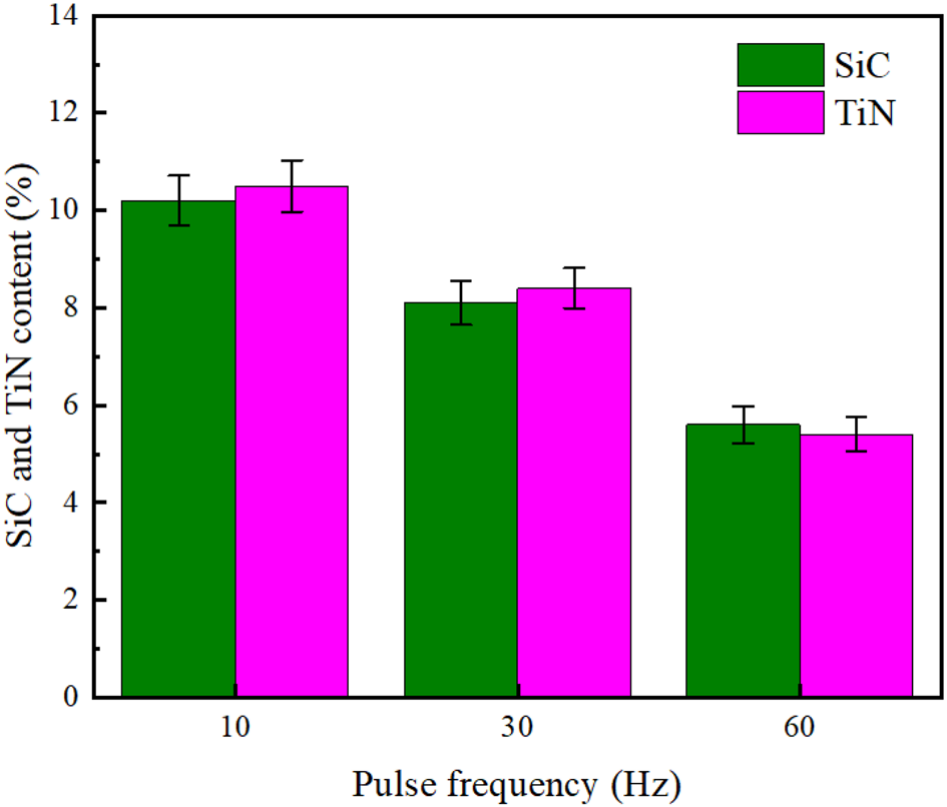
PF influence upon SiC and TiN NPs levels embedded in NCSTCCs obtained at 50% DC.

Figures 5 and 6 depict, respectively, DC/PF influence upon SiC and TiN NPs levels embedded in NCSTCCs. It was found from Fig. 5 that NCSTCCs formed at 10% DC had the smallest volume among all Ni–Co/SiC + TiN composite coatings, under fixed condition of 10 Hz PF. SiC and TiN NPs incorporated within films were measured to reveal peak SiC and TiN NP level (11.6 v/v% and 11.7 v/v%) once DC raised towards 50%. Thus far, it has been determined that higher DC conditions (i.e., 50% DC) allow for greater deposition of SiC and TiN NPs in NCSTCCs. According to the adsorption mechanism proposed by Guglielmi, the adsorption process consists of two successive stages, leading to the complete co-deposition of SiC and TiN NPs within films^18^. SiC and TiN NPs moving around the cathode were first relatively loosely adsorbed onto the electrode substrate, while metal ions completely covered the cathode surface, so that SiC and TiN NPs were deposited within the films, thereby increasing its deposition content within the films. However, the contents of SiC and TiN NPs decreased slightly when DC reached 70%. Because a high duty cycle resulted in an excessive current density, which caused the coating surface to be scorched. And part of the coating was stripped from the film, resulting in the reduction of SiC and TiN NPs contents in the film^19^.

Furthermore, under identical conditions (50% DC), as shown in Fig. 6, NCSTCCs developed through 60 Hz PF had minimal SiC and TiN contents with a volume percentage of 5.6 v/v% and 5.4 v/v% respectively. The film formed through 10 Hz PF, on the other hand, possessed peak SiC and TiN levels (10.2 v/v% and 10.5 v/v%). The variance in SiC and TiN content between films deposited at 10 Hz and 60 Hz PF, despite maintaining a constant 50% DC, can be primarily attributed to the effects of pulse frequency on the deposition kinetics and dynamics of the particles within the plating bath. At a lower pulse frequency of 10 Hz, the longer pulse duration allows for more time during each cycle for the nanoparticles to migrate and adhere to the substrate. This increased contact time enhances the likelihood of SiC and TiN nanoparticles being captured and embedded in the forming film. Conversely, at a higher frequency of 60 Hz, although the deposition cycles are more frequent, each pulse is significantly shorter. This reduction in pulse duration limits the time available for nanoparticle migration and adsorption per cycle, leading to a lower overall content of SiC and TiN nanoparticles in the deposited film. Furthermore, higher frequencies can induce more turbulent fluid dynamics in the electrolyte, potentially leading to less stable deposition conditions and reduced efficiency in nanoparticle incorporation. The experimental findings demonstrated that SiC and TiN contents within NCSTCCs exhibited a decrease as the PF was increased. Selecting an optimal PF, such as a frequency of 10 Hz, potentially drives significant over-potential across PCE depositing process, leading to enhanced energy production for the adsorption of SiC and TiN NPs onto the electrode^20^.

**Figure 7:**
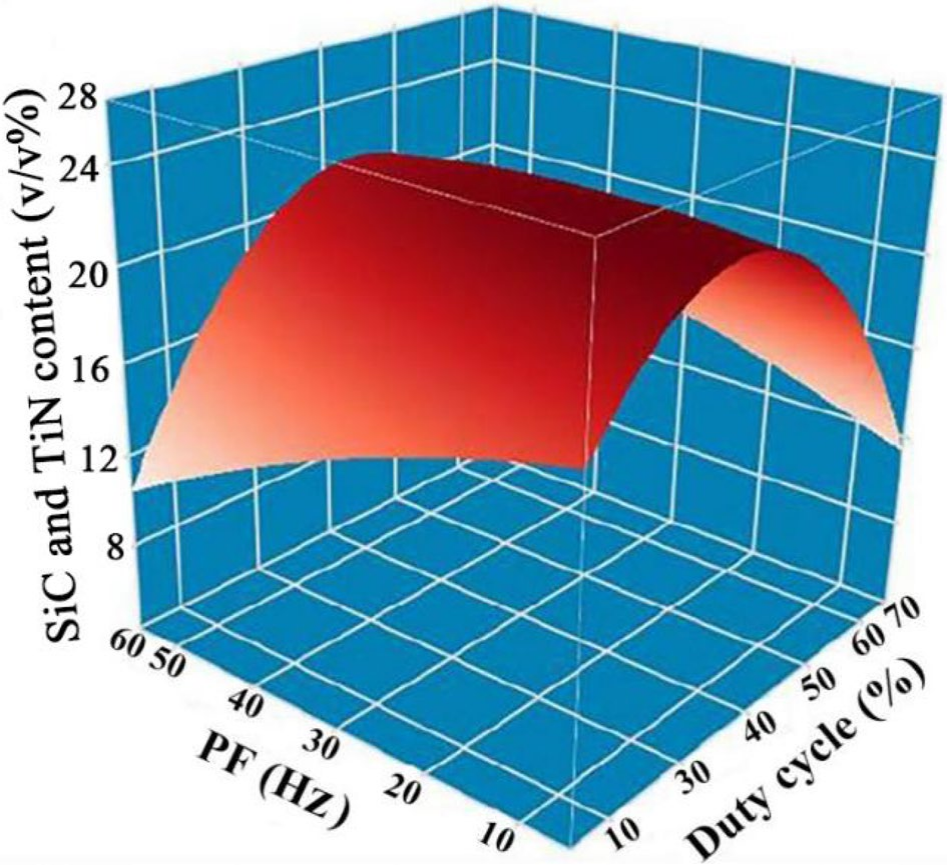
DC and PF influence upon SiC and TiN NPs levels embedded in NCSTCCs.

Figure 7 presents a three-dimensional surface diagram depicting the effects of DC and PF on the levels of SiC and TiN NPs embedded in NCSTCCs. The SiC and TiN contents in the NCSTCCs increased with an increase in DC (from 10 to 50%) and a decrease in PF (from 60 to 10 Hz). The Ni–Co/SiC + TiN composite film deposited at 50% DC and 10 Hz PF displayed the highest SiC and TiN contents among all the films. These findings were consistent with those of Figs. 5 and 6.

The increase in the embedded amount of SiC and TiN with pulse frequency (PF) can be attributed to the effect of shorter current pulses. These shorter pulses promote rapid nucleation, resulting in increased adsorption of SiC and TiN nanoparticles onto the metal surface. Furthermore, higher PF prevents nanoparticle agglomeration, enhances bath agitation, and leads to finer grain structures, which offer more surface area for nanoparticle adhesion. Consequently, the higher mobility of the nanoparticles and the frequent current pulses facilitate their incorporation into the growing composite layer, resulting in a greater amount of embedded SiC and TiN as PF increases.

### XRD characteristic analysis

**Figure 8:**
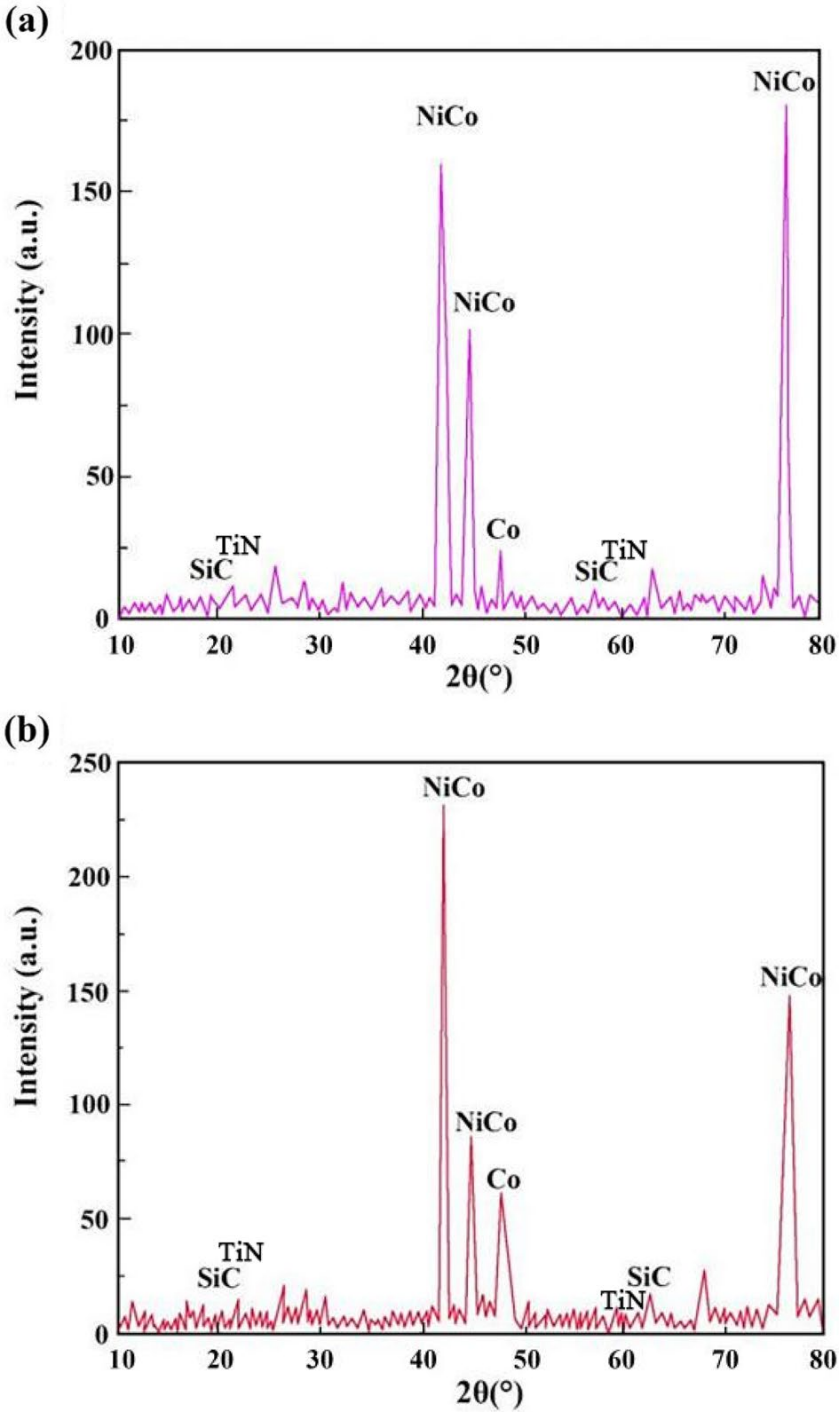
XRD patterns of NCSTCCs derived through varied DC: (**a**) 10% and (**b**) 50%.

**Figure 9:**
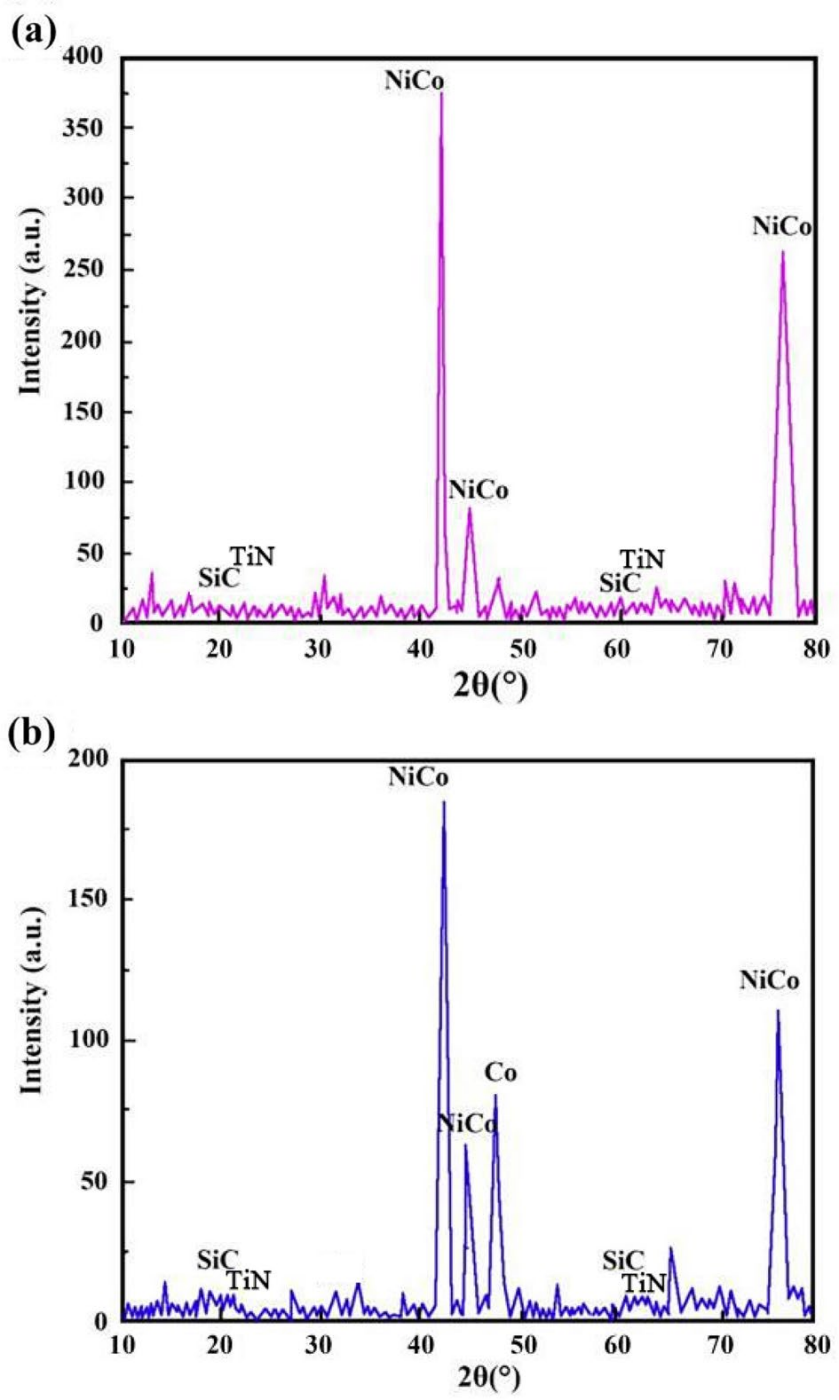
XRD patterns of NCSTCCs derived through varied PF: (**a**) 10 Hz and (**b**) 30 Hz.

Figures 8 and 9 show the XRD patterns of NCSTCCs derived through varied DC and PF variables. Weaker characteristic lines were observed when the diffraction angle of 2θ varied between 20°–40° and 50°–70°. The reason for this phenomenon was primarily associated with the fact that the density of Ni and Co was nearly three-fold that of SiC and TiN, while the fraction of SiC and TiN NPs within films was relatively low, resulting in the weak SiC (JCPDS No. 29-1129) and TiN (JCPDS No. 38-1420) peaks within the XRD patterns. Furthermore, XRD patterns exhibited the formation of NiCo with other solid mixtures containing two phases, which can be attributed to the presence of Co/Ni salts within the plating mixture. The formation of the NiCo alloy phase was confirmed by the characteristic diffraction peaks at 2θ values corresponding to the JCPDS card numbers [e.g., NiCo (JCPDS No. 45-1027)].

It is noteworthy that the strength of the characteristic diffraction peaks in composite coatings decreased with increasing PF together with reduced DC, though appearance by films’ XRD patterns did not change. Additionally, Table 2 shows the XRD spectroscopy measurements of individual grains within developed NCSTCCs. Results showed that elevating DC or decreasing PF decreased grain dimensions. When the DC and PF were kept at 50% and 10 Hz, the average NiCo size in Ni–Co/SiC + TiN nanocomposite coatings was approximately ~ 85.2 nm. Therefore, finer-grained NCSTCCs have been developed.

**Table 2:** Average NiCo sizes in NCSTCCs fabricated at varied DCs and PFs.

Plating parameters		Average NiCo size (nm)
Pulse frequency (Hz)	Duty cycle (%)	
10	10	124.2
	30	113.5
	50	85.1
	70	92.4
10	50	85.3
30		102.0
60		118.9

NCSTCCs deposited by the PCE technique are predominantly hexagonal close-packed structures (h c p) (Figs. 8 and 9), because the Ni/Co-based solid mixture within XRD spectrum reflects primarily from the (100) and (110) planes. With increasing DC (from 10 to 50%) together with reduced PF (from 60 to 10 Hz), grain size for NCSTCCs decreased, influencing SiC and TiN NPs-deposition within composite coatings. This phenomenon can be attributed to the nucleation-promoting properties of SiC and TiN NPs. The presence of SiC and TiN NPs within films led to reduced grain dimensions together with accelerating nucleation process. Additionally, the growth of grains was effectively inhibited, leading to further refinement of the grains. As previously stated, modifying DC/PF variables can result in the increase of SiC and TiN concentrations within Ni–Co/SiC + TiN composite coatings. This, in turn, has an impact on the grain size of the films during the PCE process.

### Microhardness test

**Figure 10:**
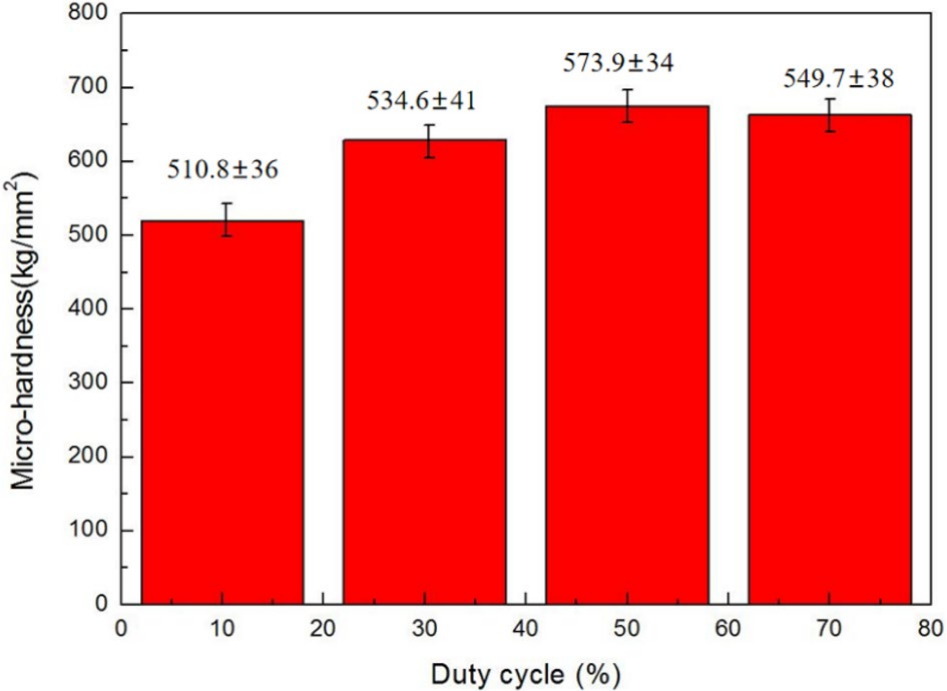
DC influence upon microhardness of NCSTCCs obtained at 10 Hz PF.

**Figure 11:**
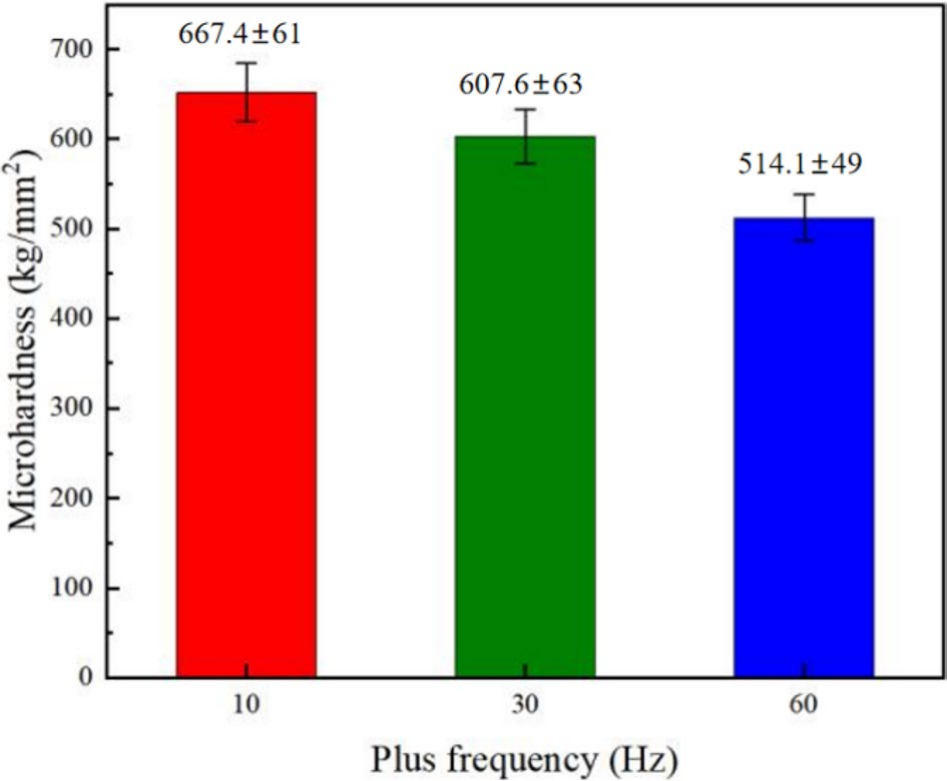
PF influence upon microhardness of NCSTCCs obtained at 50% DC.

**Figure 12:**
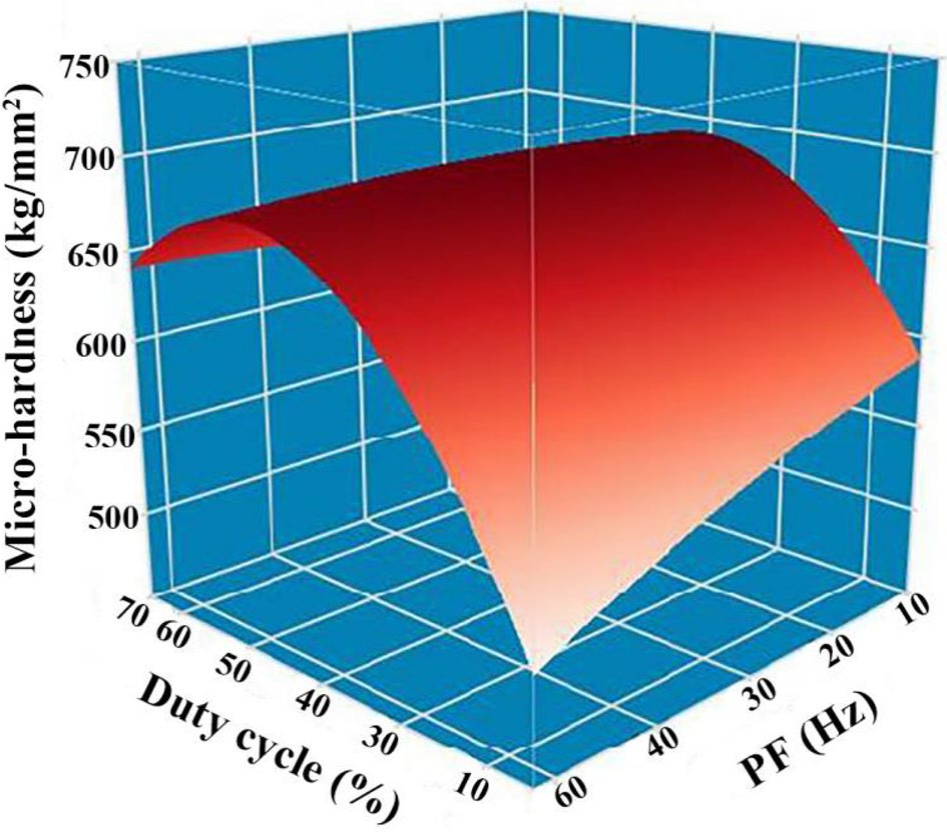
DC and PF influence upon microhardness of NCSTCCs.

Figures 10 and 11 demonstrate the microhardness values of Ni–Co/SiC + TiN composite coatings under different pulse frequencies (PFs) and duty cycles (DCs). Figure 12 shows a three-dimensional surface diagram illustrating the effects of DC and PF on the microhardness values of the films. The microhardness values of the composite coatings ranged from 510 to 670 kg/mm^2^. An increase in DC (from 10 to 50%) and a decrease in PF (from 60 to 10 Hz) resulted in an increase of microhardness value. The composite coatings prepared with a PF of 10 Hz and a DC of 50% exhibited the peak microhardness value of 667.4 kg/mm^2^. In comparison, the composite coatings prepared with a PF of 60 Hz and a DC of 10% yielded a minimal value of 514.1 kg/mm^2^.

In the co-electrodeposition process of Ni–Co/SiC + TiN composite coatings, the distribution of nanoparticles can be influenced by several factors. Non-uniform distribution is often observed due to variations in local current densities, which affect the electrochemical environment at different locations on the substrate. This variation can lead to uneven deposition rates of nanoparticles, which are further compounded by factors such as particle agglomeration and the hydrodynamic conditions within the plating bath. During our analysis, SEM images revealed areas of dense nanoparticle clusters alongside regions with fewer particles, indicating a degree of heterogeneity in particle distribution. This heterogeneity can contribute to the variability in microhardness measurements across different sample areas. Furthermore, the measured microhardness values, ranging from 510 to 670 kg/mm^2^, exhibited standard deviations that reflect the material's heterogeneous nature. Such variations underline the complex interplay between nanoparticle distribution and the composite's mechanical properties. Addressing these challenges requires careful optimization of the electrodeposition parameters and possibly the incorporation of mechanical or magnetic agitation to enhance particle dispersion.

NCSTCCs microhardness primarily depends on the amount of embedded ceramic nanoparticles (NPs) within the matrix and the fine grain structure of the composite, both of which contribute significantly to the overall hardness of the material. The microhardness of the resulting composite film is defined by the hard NPs deposited when a specific metal substrate is utilized within experiment. In this context, the contribution of the hardness of the steel substrate is minimal due to the significant thickness (~ 70 μm) of the coating. Instead, factors such as surface morphology and crystallographic orientation have a more substantial effect on microhardness. Fine grain strengthening and dispersion strengthening are the two main types of strengthening mechanisms that are determined by the size and content of NPs^21^. On the one hand, a large number of fine particles are dispersed within material strengthened by dispersion strengthening. Such fine particles obstruct dislocation movements, while matrix bears such load. On the other hand, it is demonstrated that dispersion strengthening of NPs in NCSTCCs has a significant factor for improving the microhardness of coatings^22^. The deposited NPs are uniformly dispersed within films, and the strength of Ni–Co/SiC + TiN composite coatings is improved. The cited hardness values for SiC (approximately 2840 kg/mm^2^) and TiN (approximately 3200 kg/mm^2^) are based on well-established literature and standard material properties, which illustrate the inherent hardness characteristics of these materials. These values further enhance the understanding of how such hard particles contribute to the overall hardness of the composite coating.

### EIS analysis

**Figure 13:**
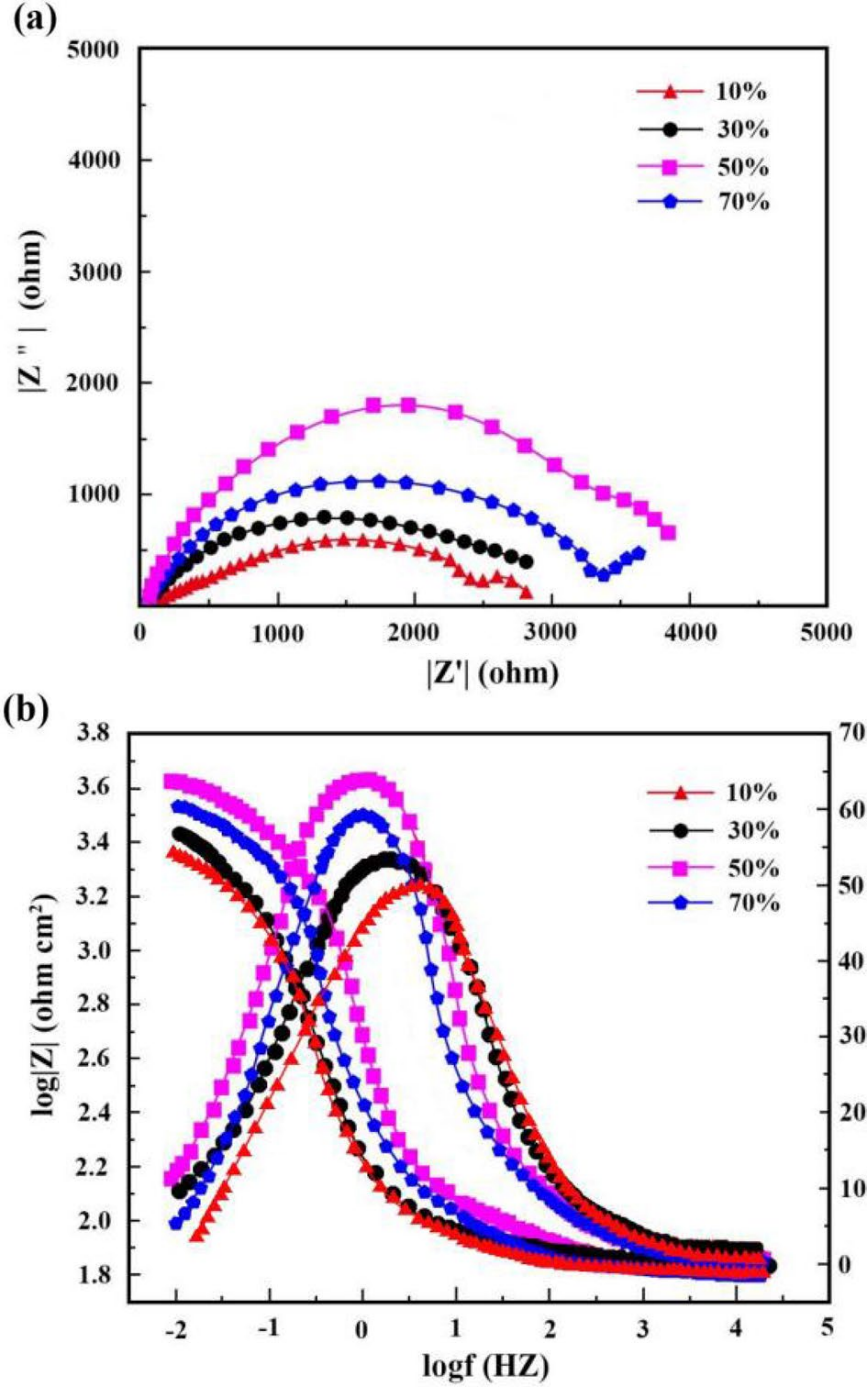
(**a**) Nyquist and (**b**) Bode curves of NCSTCCs obtained at various DC values (PF: 10 Hz).

**Figure 14:**
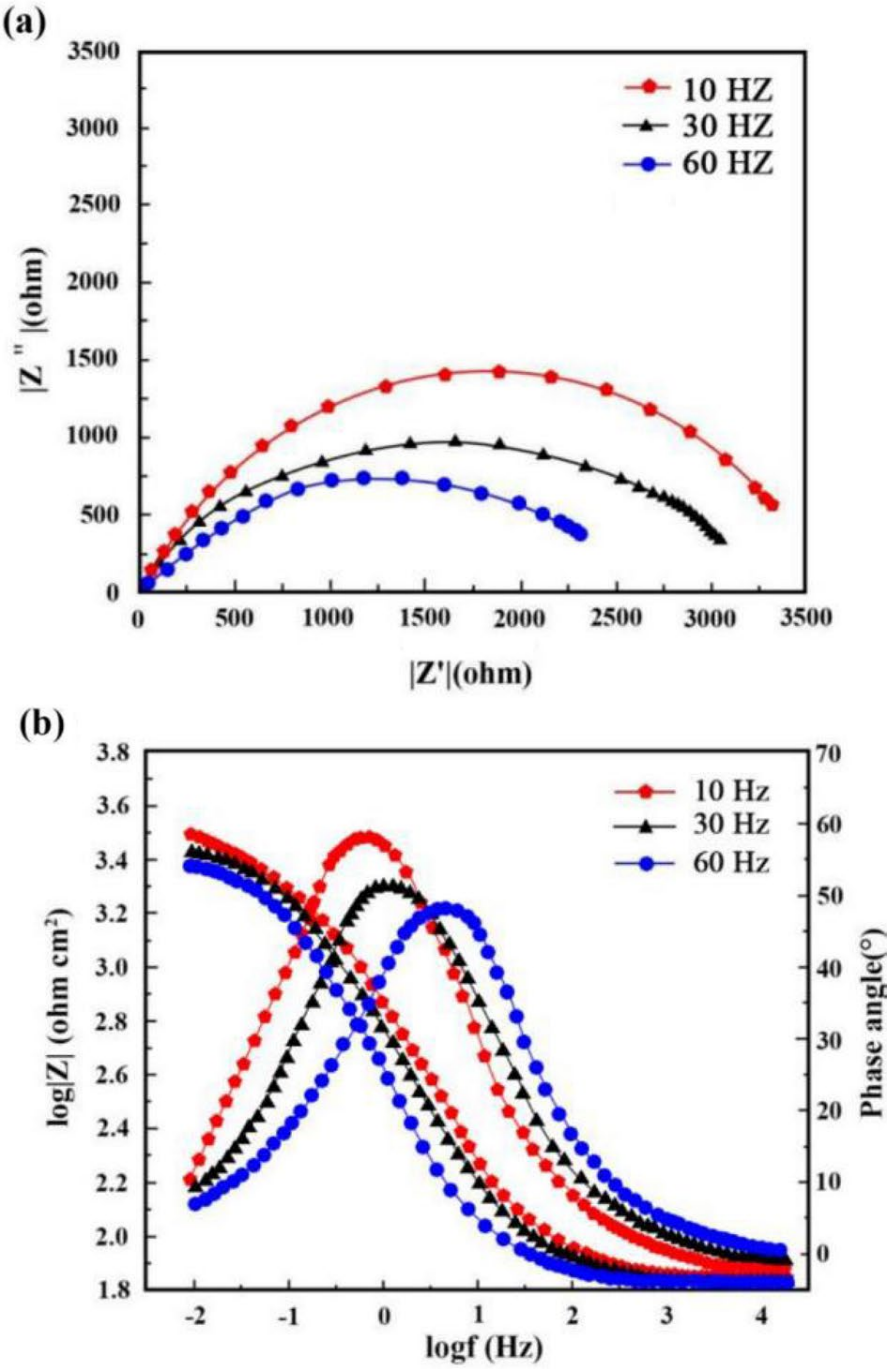
(**a**) Nyquist and (**b**) Bode curves of NCSTCCs obtained at various PF values (DC: 50%).

**Figure 15:**
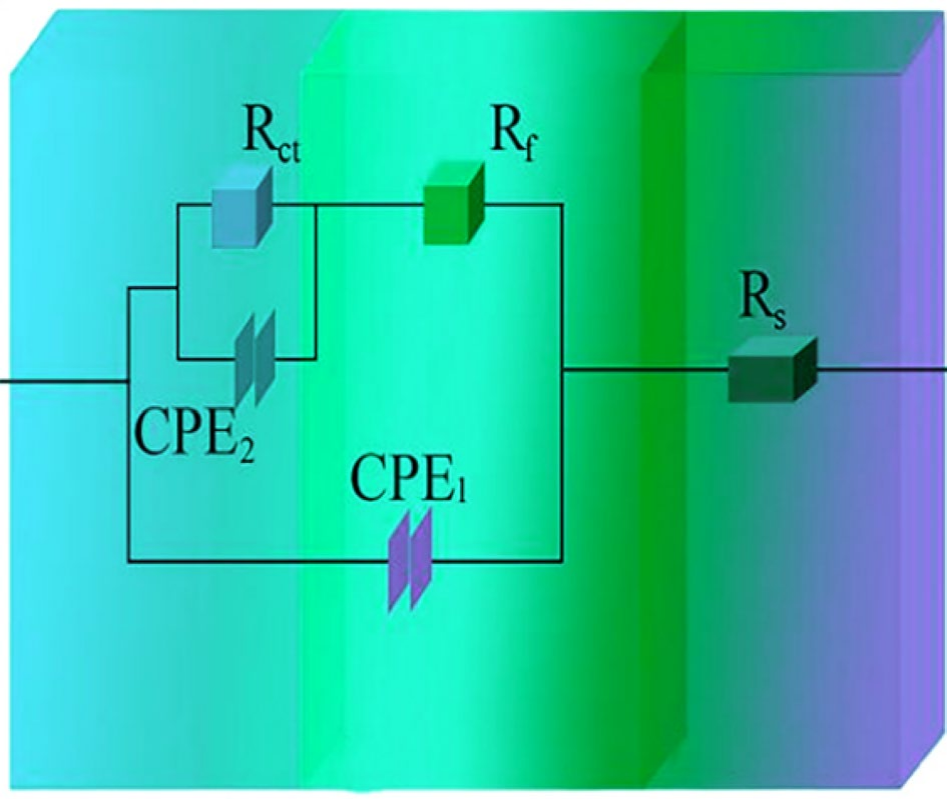
The equivalent circuit diagram in the EIS test.

Figures 13 and 14 illustrate respectively Nyquist/Bode curves for NCSTCCs attained through differing PF/DC variables. Figure 15 shows the equivalent circuit diagram in the EIS test. The high bright spots on these curves were extended across the entire frequency range using a single half-circle shape. Furthermore, as the electrode or plating bath charge was transferred, the resistance experienced a change, impacting the size of the half-circle; raising DC together with reducing PF resulted in a larger half-circle. When the DC and PF were 50% and 10 Hz, the Ni–Co/SiC + TiN composite film had the maximum charge transfer resistance (4915.7–4927.2 Ω·cm^2^), indicating an excellent corrosion resistance. This study’s findings are consistent with those of Xia and colleagues^23^.

Corrosion behavior by NCSTCCs produced under varying DC and PF electrodeposition settings was investigated using an analogous circuit. This equivalent circuit model contains charge transfer resistor and a mixture resistor linked in parallel, together with a double-charge layer capacitor used to assess electrochemical parameters. Table 3 illustrates influence from DC/PF variables upon charge transfer resistance, which is inversely related to corrosion rate. Obviously, since DC was raised, the resistance steadily increased, implying that the corrosion rate of manufactured composite coatings decreased faster while corrosion resistance was excellent. The corrosion rate slowed down when the PF was steadily decreased, further confirming the greater resistance of composite coatings to corrosion.

**Table 3:** Charge-transfer resistances collected from EIS plots on Ni–Co/SiC + TiN composite coatings.

Plating parameters		Charge transfer resistance (Ω·cm^2^)
Pulse frequency (Hz)	Duty cycle (%)	
10	10	2901.4
	30	3654.3
	50	4915.7
	70	4297.6
10	50	4927.2
30		3416.9
60		2479.7

Strength, hardness, and structural stability for NCSTCCs were considerably improved through deposition of SiC and TiN NPs, which may further improve the films’ corrosion properties^24^. In addition, presence of numerous SiC and TiN NPs upon cathode surface of films reduces functional regions concerning such reduction reaction occurring upon cathode surface, thereby decreasing the anode dissolution rate.

### Potentiodynamic polarization test analysis

**Figure 16:**
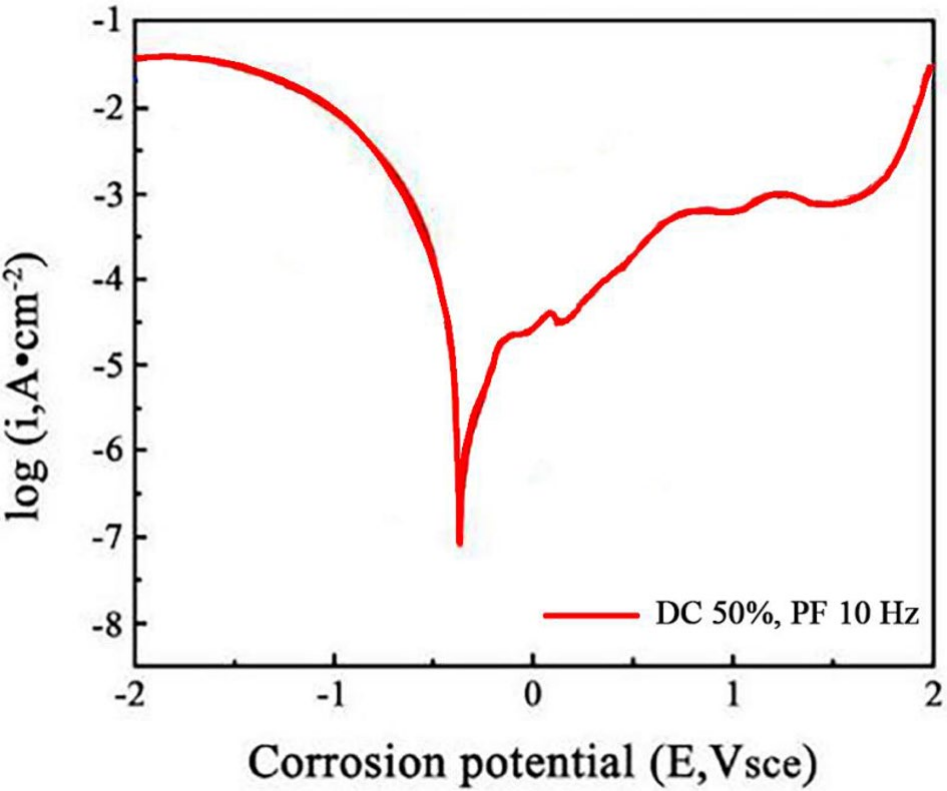
The polarization curves at a duty cycle (DC) of 50% and a pulse frequency (PF) of 10 Hz.

**Figure 17:**
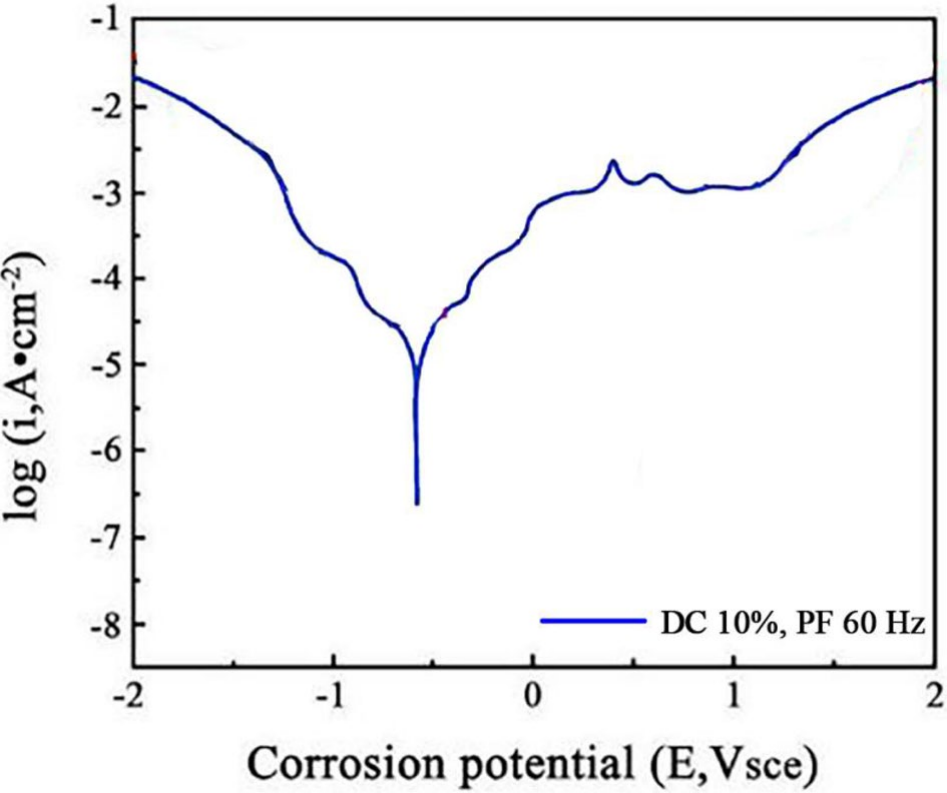
The polarization curves at a duty cycle (DC) of 10% and a pulse frequency (PF) of 60 Hz.

To further investigate the corrosion resistance of the Ni–Co/SiC + TiN composite coatings (NCSTCCs), a potentiodynamic polarization (PDP) test was conducted. The polarization curves are presented in Figs. 16 and 17. The test was carried out using an electrochemical workstation in a seawater-like solution (NaCl 25 g/L, MgSO_4_ 3 g/L, MgCl_2_ 2 g/L, CaCl_2_ 1 g/L) with a platinum electrode as the counter electrode and a saturated calomel electrode (SCE) as the reference electrode.

The obtained polarization curves were analyzed to derive corrosion potential (Ecorr) and corrosion current density (Icorr). The corrosion current density was used to calculate the corrosion rate of the films, providing a quantitative measure of corrosion resistance, which was calculated by using Eq. (1). The results indicate that the NCSTCCs prepared at a duty cycle (DC) of 50% and a pulse frequency (PF) of 10 Hz exhibited the lowest corrosion current density (1 × 10^−6^ A/cm^2^) and the most positive corrosion potential (− 0.45 V vs. SCE), indicating the highest corrosion resistance (3.68 mm/year). In contrast, the films produced at 10% DC and 60 Hz PF showed a higher corrosion current density (1 × 10^−5^ A/cm^2^) and more negative corrosion potential (− 0.55 V vs. SCE), reflecting a reduced corrosion resistance (18.49 mm/year).

1 $$CR=\frac{K\times {I}_{coorr}\times Eq.Weight}{\rho \times A}$$

where *K* denotes the constant that depends on the unit system, where ${I}_{coorr}$ refers to corrosion current density, where $Eq.Weight$ represents the equivalent weight of the material being tested, where $\rho$ expresses the density of the material and *A* represents the area of the exposed surface.

These findings are consistent with the results from the Electrochemical Impedance Spectroscopy (EIS) tests, confirming that the corrosion resistance improves with increasing DC and decreasing PF. The presence of SiC and TiN nanoparticles in the NCSTCCs contributes to this enhancement, as they provide barrier effects, refine the grain size, and improve the passivation behavior.

## Conclusions

1. Ni–Co/SiC + TiN composite coatings exhibited cauliflower-like globular morphological structures. The cauliflower-like structural morphology observed at low-DCs changed to the nodular morphology observed at high-DCs, in which the cauliflower-like grains became small and fine. SiC and TiN NPs were placed within a matrix containing NCSTCCs as second phases.
2. NCSTCCs made under 10% DC exhibited minimal SiC and TiN content with a percent volume of just 5.6 v/v% and 5.4 v/v% respectively under the fixed condition of 60 Hz PF. However, the composite coatings were developed at a 50% DC, resulting within highest SiC and TiN concentration with a percent volume of 11.6 v/v% and 11.7 v/v% respectively.
3. When the 2θ diffraction angle was between 20°–40° and 50°–70°, weaker SiC and TiN characteristic lines were observed in NCSTCCs due to a limited amount of SiC and TiN deposition. Furthermore, decreasing the PF or raising DC parameters would reduce grain size of composite coatings.
4. NCSTCCs developed at 50% DC together with 10 Hz PF achieved a microhardness of 667.4 kg/mm^2^, while nanocomposite coatings achieved a microhardness of 514.1 kg/mm^2^ once prepared using 10% DC and 60 Hz PF. When the DC and PF were 50% and 10 Hz, the Ni–Co/SiC–TiN composite film had the maximum charge transfer resistance (4915.7–4927.2 Ω·cm^2^), indicating an excellent corrosion resistance.

## Data Availability

The datasets used and/or analysed during the current study available from the corresponding author on reasonable request.
